# Long-term survival analysis in combined transarterial embolization and stereotactic body radiation therapy versus stereotactic body radiation monotherapy for unresectable hepatocellular carcinoma >5 cm

**DOI:** 10.1186/s12885-016-2894-9

**Published:** 2016-11-03

**Authors:** Ting-Shi Su, Huan-Zhen Lu, Tao Cheng, Ying Zhou, Yong Huang, Ying-Chuan Gao, Min-Yang Tang, Hua-Yan Jiang, Zu-Ping Lian, En-Cun Hou, Ping Liang

**Affiliations:** 1 Department of Radiation Oncology, Rui Kang Hospital, Guangxi Traditional Chinese Medical University, Nanning, 530001 Guangxi Zhuang Autonomous Region China; 2 Cyberknife Center, Rui Kang Hospital, Guangxi Traditional Chinese Medical University, Nanning, 530001 Guangxi Zhuang Autonomous Region China; 3 Department of Medical Oncology, Rui Kang Hospital, Guangxi Traditional Chinese Medical University, Nanning, 530001 Guangxi Zhuang Autonomous Region China

**Keywords:** Hepatocellular carcinoma, HCC, TAE/TACE, Stereotactic body radiation therapy, SBRT

## Abstract

**Background:**

The survival following transarterial chemoembolization (TACE) alone is still low in unresectable hepatocellular carcinoma (HCC) with almost patients developing disease progression after treatment. There is need to investigate additional therapeutic options that would intensify the initial response to TACE. The present study was to retrospectively compare the outcome and evaluate the prognostic factors of stereotactic body radiation therapy (SBRT) alone or as an adjunct to transarterial embolization (TAE) or TACE in the treatment of HCC >5 cm.

**Methods:**

From January 2011 to April 2015, 77 patients received SBRT followed by TAE or TACE (TAE/TACE + SBRT group) and 50 patients received SBRT alone (SBRT group). The dose of SBRT was 30–50 Gy which was prescribed in 3–5 fractions. Eligibility criteria were: a longest tumor diameter >5.0 cm and Child-Turcotte-Pugh (CTP) Class A or B. Exclusion criteria included tumor thrombus, lymph node involvement and extrahepatic metastasis.

**Results:**

The median follow-up period was 20.5 months. Median tumor size was 8.5 cm (range, 5.1–21.0 cm). Median overall survival (OS) in the TAE/TACE + SBRT group was 42.0 months versus 21.0 months in the SBRT group. The 1-, 3- and 5-year OS was 75.5, 50.8, and 46.9 % in the TAE/TACE + SBRT group and was 62.4, 32.9, and 32.9 % in the SBRT group, respectively (*P* = 0.047). The 1-, 3- and 5-year distant metastasis-free survival (DMFS) was 66.3, 44.3, and 40.6 % in the TAE/TACE + SBRT group and was 56.8, 26.1, and 17.4 % in the SBRT group, respectively (*P* = 0.049). The progression-free survival (PFS) and local relapse-free survival (LRFS) were not significantly different between the two groups. In the entire patient population, a biologically effective dose (BED_10_) ≥100 Gy and an equivalent dose in 2 Gy fractions (EQD_2_) ≥74 Gy were significant prognostic factors for OS, PFS, LRFS and DMFS.

**Conclusions:**

SBRT combined with TAE/TACE may be an effective complementary treatment approach for HCC >5 cm in diameter. BED_10_ ≥100 Gy and EQD_2_ ≥74 Gy should receive more attention when the SBRT plan is designed.

## Background

According to global cancer statistics, an estimated 782,500 new liver cancer cases and 745,500 deaths occurred worldwide in 2012, with China alone accounting for approximately 50 % of the total number of cases and deaths [1]. The main histological subtype of primary liver cancer occurring worldwide is hepatocellular carcinoma (HCC) [1]. Resection or transplantation is the gold standard for the treatment of early-stage HCC [2]. However, only 10–20 % of newly diagnosed patients have resectable disease. The majority of HCCs are unresectable or non-transplantable at the time of diagnosis. Transarterial chemoembolization (TACE) is frequently used as a local treatment option for unresectable or non-transplantable HCC, which imparts a survival benefit compared to best supportive care [3, 4].

Traditionally, radiotherapy (RT) has played a limited role in the treatment of HCC due to radiation-induced liver disease (RILD) and low tolerance of the whole liver to irradiation with a dose of 30–35 Gy [5]. Recently, stereotactic body radiation therapy (SBRT) has been investigated as a research hotspot, in order to provide a higher biologically effective dose (BED). Encouraging results have indicated that liver SBRT is safe with a high rate of local control [6–10].

In the present study, we aimed to retrospectively compared the long-term survival in combined transarterial embolization (TAE/TACE) and SBRT, and SBRT alone for unresectable HCC >5 cm in diameter at our institution.

## Methods

### Patient population

From January 2011 to April 2015, 127 patients with unresectable HCC were treated with SBRT alone or as an adjunct to TAE/TACE. Eligibility criteria included: (a) primary HCC was diagnosed by surgeon, and/or radiologist and oncologist, according to the international guidelines for the management of HCC or by pathology [2], (b) longest tumor diameter >5.0 cm, (c) Child-Turcotte-Pugh (CTP) Class A or B disease, and (d) Eastern Cooperative Oncology Group (ECOG) score 0–1. Exclusion criteria were: (a) tumor thrombus, (b) lymph node involvement and extrahepatic metastasis, (c) ECOG ≥2, and (d) poor liver function in CTP C disease.

Patients without increased CTP score and hepatic enzyme (ALT or/and AST) higher than normal before TAE/TACE, were treated with SBRT after TAE/TACE following an interval of 3 to 4 weeks. Patients with a hepatic arteriovenous fistula or who refused to undergo TAE/TACE received SBRT only. Patient characteristics in the TAE/TACE + SBRT group and the SBRT group are shown in Tables 1 and 2. All patients provided written informed consent. Ethical approval was obtained from the Medical Ethics Committee of Rui Kang Hospital, Guangxi, China.

**Table 1:** Baseline characteristics of the TACE/TAE+ SBRT and SBRT groups

	TACE/TAE + SBRT (*n* = 77)	SBRT (*n* = 50)	*P* value
Age ≥60/<60 years	60/17	39/11	0.992
Gender (male/female)	67/10	45/5	0.610
HBsAg (unknown/negative/positive)	0/4/73	2/4/44	0.456
CTP class (A/B)	70/7	41/9	0.139
ECOG (0/1)	60/17	39/11	0.545
Nodules (solitary/multiple)	50/27	37/13	0.283
Recurrent/Primary	10/67	5/45	0.324
BCLC (A/B)	50/27	37/13	0.283
Liver cirrhosis (Yes/No)	57/20	38/12	0.485
AFP ≥100 ng/mL (Yes/No)	44/33	30/20	0.562
Tumor size (5–10 cm/≥10 cm)	51/26	43/7	0.013*
EQD_2_ (≥/<74Gy)	49/28	43/7	0.150
BED_10_ (≥/<100Gy)	24/53	21/29	0.031*
30-45Gy/3Fr;38-48Gy/4Fr;35-50Gy/5Fr	43/23/11	31/15/4	0.695

*AFP* alpha fetoprotein, *BED* biologically effective dose, *BCLC* Barcelona clinic liver cancer, *CTP* Child–Turcotte–Pugh, *EQD*_*2*_ equivalent dose in 2Gy fraction, *ECOG* Eastern cooperative oncology group, *Fr* fractions, *SBRT* stereotactic body radiation therapy, *TACE/TAE* trans-arterial embolization; *P < 0.05

**Table 2:** Univariate analysis and multivariate analysis of prognostic predictors for OS and DMFS in 127 locally unresectable HCC patients

Characteristics		Patients	OS (%)		Univariate analysis	Multivariate analysis	DMFS (%)		Univariate analysis	Multivariate analysis
			1-year	3-year			1-year	3-year		
Gender	Male	112	71.1	46.3	0.275		63.1	38.8	0.568	
	Female	15	66.7	34.6			60.0	35.0		
Age, years	≥60	29	69.9	45.1	0.740		68.7	48.5	0.558	
	<60	98	70.9	43.2			61.2	35.9		
AFP ≥100 ng/mL	Yes	74	63.4	33.4	0.009*	0.057	55.8	28.2	0.022*	0.081
	No	53	80.3	61.1			70.2	52.5		
BCLC stage	A	87	67.5	45.0	0.924		60.6	37.8	0.934	
	B	40	76.9	44.6			66.8	39.5		
CTP class	A	111	74.0	45.3	0.112		66.1	39.4	0.099	
	B	16	60.6	44.6			36.1	27.0		
TACE/TAE	Yes	77	75.5	50.8	0.047*	0.017*	66.3	44.3	0.049*	0.011*
	No	50	62.4	32.9			56.8	26.1		
BED_10_ (Gy)	≥100	44	87.8	62.0	0.005*	0.049*	80.2	50.2	0.006*	0.023*
	<100	83	61.4	35.4			53.3	30.6		
Tumor size (cm)	5–10	94	77.2	47.3	0.048*	0.137	67.2	42.0	0.064	0.053
	≥10	33	51.8	39.1			49.8	28.9		
EQD_2_ (Gy)	≥74	88	75.4	52.0	0.046*	0.051	68.0	46.3	0.008*	0.035*
	<74	39	59.9	28.1			51.2	21.6		

*CTP* Child–Turcotte–Pugh, *SBRT* stereotactic body radiation therapy, *AFP* alpha fetoprotein, *BED* biologically effective dose, *EQD*_*2*_ equivalent dose in 2Gy fraction, *OS* overall survival, *DMFS* distant metastasis-free survival, *TACE/TAE* trans-arterial embolization; *P < 0.05

### TAE/TACE

TAE procedure: selective arteriography of the hepatic artery was performed to locate the tumor. After identifying the tumor-feeding artery, 5–20 mL Lipiodol (Huai Hai Pharmaceutical Factory, Shanghai, China) was slowly injected through the catheter, which was followed by gelatin sponge particle Gelfoam (Jinling Pharmaceutical Co., Ltd., Nanjing, China) embolization. TACE procedure: selective arteriography of the hepatic artery was performed to locate the tumor. After identifying the tumor-feeding artery, a mixture of 5–20 mL Lipiodol (Huai Hai Pharmaceutical Factory, Shanghai, China) and 30–40 mg/m^2^ cisplatinum or fluorouracil glycosides 750–1000 mg was slowly injected through the catheter, which was followed by gelatin sponge particle Gelfoam (Jinling Pharmaceutical Co., Ltd., Nanjing, China) embolization. TAE/TACE was repeated 1 to 4 times at intervals of 4 to 6 weeks.

### SBRT

Three or four gold markers (0.8 mm in diameter) were implanted into the tumor guided by B-ultrasound or CT. One week later, CT scan and MRI were performed with a slice thickness of 3 mm. Image fusion CT and MRI delineated the gross tumor volume (GTV) and was expanded by 0–3 mm to form the planning target volume (PTV). CyberKnife Robotic Radiosurgery (Accuray Inc., Sunnyvale, CA, USA) involved use of the Synchrony respiratory motion-tracking system and patients wore a special Synchrony vest. The average treatment time was 30–60 min. A dose of 30–50 Gy was prescribed in 3–5 fractions at the 66 % (range, 56–80 %) isodose line.

The dose–volume constraints for organs at risk (OARs) were: duodenum: V 1 mL <25 Gy; stomach and small bowel: V 1 mL <25 Gy; kidneys: 1/3 V_total_ <15 Gy; liver: total spared volume (V_total_-V_15Gy_) >700 mL and/or V_15Gy_ <1/3V_total_; and spinal cord: V 1 mL <15 Gy.

The CyberKnife platform utilized 95–230 beams. Maximum spinal cord point dose was a mean of 6.5 Gy (range, 4.2–12.9 Gy), which was strictly maintained at 1 mL <15 Gy. Maximum bowel point dose was a mean of 21.2 Gy (range, 8–29.9 Gy) for the PTV, which was strictly maintained at 1 mL <25 Gy. Liver total spared volume: V _total_-V_15Gy_ was a mean of 810 mL (range, 600–1330 mL) and/or V_15Gy_ <1/3V_total_. According to the standard equation, the biologically effective dose (BED) and equivalent dose in 2 Gy fractions (EQD_2_) assumed an α/β ratio of 10 for rapidly proliferating tumor cells and 3 for normal tissues.

$$ \mathrm{BED} = \mathrm{d} \times \mathrm{n}\left\{1 + \mathrm{d}/\left(\upalpha /\upbeta \right)\right\};\ \mathrm{E}\mathrm{Q}\mathrm{D} = \mathrm{d} \times \mathrm{n}\left\{\left(\upalpha /\upbeta + \mathrm{d}\right)/\left(\upalpha /\upbeta + \mathrm{d}\mathrm{x}\right)\right\};\ \left(\mathrm{d} = \mathrm{d}\mathrm{ose},\ \mathrm{n} = \mathrm{fraction}\ \mathrm{and}\ \mathrm{d}\mathrm{x} = 2\right). $$

### Adjuvant therapy

During SBRT treatment, combined adjuvant medication was administered, consisting of lansoprazole, glutathione, vitamins and Chinese herbs. Antiviral therapy with a nucleoside or nucleotide analogue was also administered to patients with chronic hepatitis due to hepatitis virus B (HBV) infection.

### Response evaluation and follow-up

This study was censored on August 1, 2016. Patients were re-evaluated one month after SBRT and every three months thereafter by the treating radiation oncologist. Clinical examination, determination of alpha fetoprotein (AFP) and contrast-enhanced CT and/or MRI were performed at each follow-up visit. The Response Evaluation Criteria in Solid Tumors (RECIST) guideline was used to describe changes in the treated areas [11].

### Toxicity

Toxicity induced by SBRT was scored according to the NCI Common Terminology Criteria for Adverse Events (CTCAE) version 4.03. RILD was defined as an anicteric elevation in alkaline phosphatase of at least 2-fold the upper normal level (classic RILD) or elevated transaminases of at least 5-fold (non-classic RILD), without progressive disease (PD) and the development of nonmalignant ascites [12]. Treatment-associated complications were scored according to the NCI CTCAE version 4.03. To verify late adverse effects on liver function, deterioration in the CTP score after 6 months was also determined in evaluable patients, defined as alive without PD or intrahepatic recurrence, and having received no additional therapy in the 6 months after completion of SBRT.

### Statistical analysis

SPSS version 17.0 (IBM Corp., Armonk, NY, USA) was used for statistical analysis. A Kaplan–Meier curve was used to calculate the OS, PFS, LRFS, and DMFS rates. OS was calculated from the date of SBRT treatment until the date of final follow-up or death. PFS was estimated from the date of SBRT treatment until the date of disease progression or death. LRFS was estimated from the date of SBRT treatment until the date of intrahepatic recurrence or death. DMFS was estimated from the date of SBRT treatment until the date of extrahepatic metastases or death. The log-rank test was used to compare outcomes among survival curves for each potential prognostic factor. Any factors that were significant in univariate analyses were subjected to multivariate analyses using the Cox proportional hazards regression model. For comparisons between the baseline variables, the *X* ^2^ test and Fisher’s exact test were performed. *P* < 0.05 was considered statistically significant.

## Results

### Patient characteristics

In total, 77 patients received TAE/TACE + SBRT and 50 patients received SBRT alone in Rui Kang Hospital, Nanning, China. Median patient age was 51 years (range, 21–86 years) and 112 patients were male. One hundred and eleven patients (86.4 %) had CTP Class A disease. Eighty-seven patients had Barcelona Clinic Liver Cancer (BCLC) A stage disease. Thirty-three patients had HCC with the largest diameter ≥10 cm. Patient characteristics are shown in Tables 1 and 2.

### TAE/TACE + SBRT versus SBRT alone

**Fig. 1:**
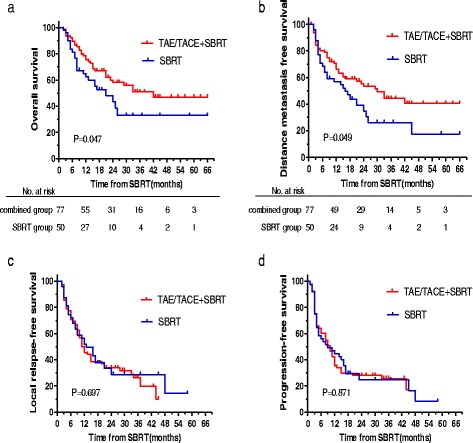
TAE/TACE + SBRT group versus SBRT group. **a** OS; **b** DMFS; **c** LRFS; **d** PFS

During the follow-up period, 58 patients (45.7 %) died (Table 3). Median OS in the TAE/TACE + SBRT group was 42.0 months versus 21.0 months in the SBRT group. The 1-, 3- and 5-year OS was 75.5, 50.8, and 46.9 % in the TAE/TACE + SBRT group and was 62.4, 32.9, and 32.9 % in the SBRT group, respectively (*P* = 0.047). The 1-, 3- and 5-year DMFS was 66.3, 44.3, and 40.6 % in the TAE/TACE + SBRT group and was 56.8, 26.1, and 17.4 % in the SBRT group, respectively (*P* = 0.049). PFS and LRFS were not significantly different between the two groups (Fig. 1).

**Table 3:** Causes of death analysis in the two groups

Characteristics	TAE/TACE + SBRT group	SBRT group
Cases of death	33	25
Causes of death		
Tumor-related	30	21
Non tumor-related	3	4
Local tumor-related	15	6
Metastasis-related	15	15
Cases of death within 6 months	8	9
Causes of death within 6 months		
Radiation-induced liver disease	2	1
Tumor progression	6	8

*SBRT* stereotactic body radiation therapy, *TACE/TAE* transarterial embolization

### Toxicity

The most common toxicities were grade ≤2 (Table 4). In SBRT group, RILD was observed in one patient with CTP B who died 3 months after SBRT treatment. Five patients experienced grade 3/4 toxicity in the SBRT group. No acute grade 5 toxicities were observed. Four of these patients recovered with conservative treatment. After 6 months, worsening of CTP score by one points was observed in one A5, and one B7 patient. Worsening of CTP score by two points was observed in one B7 patient.

**Table 4:** Toxicity in the two groups after SBRT in 6 months

Toxicity (Grade)	TAE/TACE + SBRT group			SBRT group			*P* value
	1	2	≥3	1	2	≥3	
Epigastric discomfort	5	1	1	4	0	1	
Nausea	3	1	0	2	1	0	
Vomiting	2	1	2	1	0	1	
Anemia	2	0	0	2	1	0	
Gastric ulcer	0	0	1	0	0	1	
Fatigue	4	2	0	3	1	0	
Liver failure	0	0	2	0	0	1	
Gastrointestinal hemorrhage	0	0	1	0	0	1	
Total	16/77	5/77	7/77	12/50	3/50	5/50	0.442

*ALT* alanine aminotransferase, *AST* aspartate aminotransferase, *SBRT* stereotactic body radiation therapy, *TACE/TAE* transarterial embolization

In TAE/TACE + SBRT group, RILD was observed in one CTP A patient and one CTP B patient. Six patients experienced grade 3/4 toxicity and one patient experienced grade 5 liver failure. Six of these patients recovered with conservative treatment. After 6 months, worsening of CTP score by one points was observed in two A5, one A6 and one B7 patients. worsening of CTP score by two points was observed in one A5 patient.

### OS, PFS, LRFS, and DMFS

The median follow-up period was 20.5 months (range, 2–66 months) in the entire patient population. Median tumor size was 8.5 cm (range, 5.1–21.0 cm). The median OS was 26.0 months and the 1-, 3- and 5-year OS was 70.5, 44.8, and 42.0 %, respectively. The median DMFS was 23.0 months and the 1-, 3- and 5-year DMFS was 65.4, 38.2, and 32.1 %, respectively. The median PFS was 11.0 months and the 1- and 3-year PFS was 39.2 and 25.8 %, respectively. The median LRFS was 12.0 months and the 1- and 3-year LRFS was 48.1 and 26.8 %, respectively.

### Prognostic factors for OS and DMFS

Prognostic factors evaluated by univariate and multivariate analyses included gender, age, AFP, BCLC stage, CTP class, BED_10_, EQD_2_, tumor size and TAE/TACE (Table 4).

**Fig. 2:**
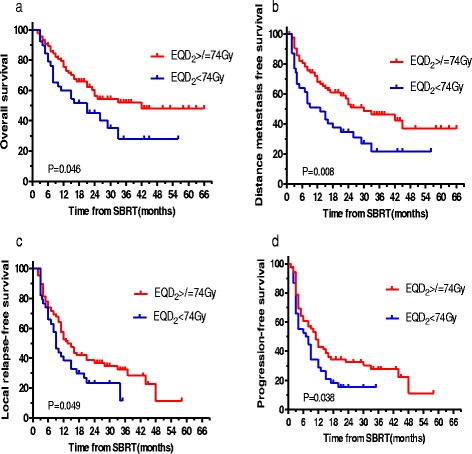
EQD_2_ ≥74 Gy was a significant prognostic factor. **a** OS; **b** DMFS; **c** LRFS; **d** PFS

**Fig. 3:**
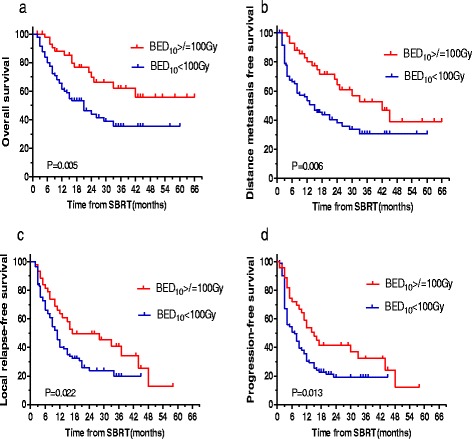
BED_10_ ≥100 Gy was a significant prognostic factor. **a** OS; **b** DMFS; **c** LRFS; **d** PFS

Univariate analysis with log-rank test identified five significant prognostic factors for OS: TAE/TACE, tumor size, EQD_2_ (Fig. 2a), BED_10_ (Fig. 3a) and AFP ≥100 ng/mL. Multivariate analysis using the Cox model identified two significant prognostic factors for OS: TAE/TACE and BED_10_ (all *P* < 0.05).

Univariate analysis with the log-rank test identified four significant prognostic factors for DMFS: TAE/TACE, EQD_2_ (Fig. 2b), BED_10_ (Fig. 3b) and AFP ≥100 ng/mL. In addition, TAE/TACE, BED_10_, and EQD_2_ were significant prognostic factors for DMFS when each continuous variable was entered individually into the Cox model (*P* < 0.05).

## Discussion

HCC is a highly prevalent disease in many Asian countries, accounting for 80 % of cases worldwide. Less than 20 % of HCC patients are surgical candidates at diagnosis [13]. TACE is frequently used as a local treatment option for unresectable or non-transplantable HCC, which imparts a survival benefit compared to best supportive care [3, 4]. The BCLC staging system recommends TACE as the standard treatment for intermediate stage disease. Tumor response rate to TACE ranged from 17 to 61.9 %, but complete tumor response is very low (0–4.8 %) as relapse occurs due to intracapsular or extracapsular invasion by HCC. The long-term survival rates are very low for TACE (5-year OS:1–8 %) [14]. Local treatments for unresectable HCC commonly used in combination with TAE/TACE for unresectable HCC are thermal ablation, percutaneous ethanol injection, and radiation therapy in Asian countries [13, 15]. Numerous clinical studies have reported improved outcomes using TACE + RT combination therapy compared with TACE [16–18] or RT alone [19]. SBRT has been investigated as a research hotspot in order to provide a higher BED. Encouraging results have indicated that SBRT is a safe therapy with a high rate of local control in patients with HCC [10, 20–22].

Combined TACE and SBRT may be a new treatment approach for unresectable HCC. Honda et al. retrospectively evaluated 28 patients with small HCCs treated with TACE followed by SBRT. The median disease-free survival time was 18 months. The local control (LC) and OS at 1 year was 96.3 and 92.6 %, respectively. None of the patients experienced severe acute hematological or physical toxicity or radiation-induced liver damage [23]. Kang et al. reported the findings of a phase 2 trial of HCC patients (median longest diameter 29 mm, range 13–78 mm) treated with SBRT after incomplete TACE. All patients underwent TACE 1–5 times before SBRT. SBRT doses ranged from 42 to 60 Gy in 3 fractions and complete response was 38.3 % at 6 months. The LC, OS and PFS at 2 years were 94.6, 68.7 and 33.8 %, respectively. In addition, 6.4 % of patients experienced grade 3 gastrointestinal toxicity and 4.3 % of patients experienced grade 4 gastric ulcer perforation [24]. Jacob et al. reported that local recurrence was significantly decreased in the TACE + SBRT group compared with the TACE only group (10.8 % versus 25.8 %, *P* = 0.04). After censoring for liver transplantation, OS was significantly increased in the TACE + SBRT group compared with the TACE only group (33 months versus 20 months, *P* = 0.02) [25]. In the present study, we report long-term survival following combined TAE/TACE and SBRT versus SBRT alone for unresectable HCC >5 cm. Our previous study demonstrated that SBRT is an alternative treatment for small (≤5 cm) HCC, with OS at 1, 3, and 5 years of 94.1, 73.5, and 64.3 %, respectively [26]. In the current study, median OS in the TAE/TACE + SBRT group was better than that in the SBRT only group. The 1-, 3- and 5-year OS was 75.5, 50.8, and 46.9 % in the TAE/TACE + SBRT group and was 62.4, 32.9, and 32.9 % in the SBRT group, respectively (*P* = 0.047). Therefore, SBRT combined with TAE/TACE may be an effective complementary treatment approach for HCC >5 cm.

Radiation dose and fractions varied during SBRT. EQD_2_ ≥74 Gy (Fig. 2) and BED_10_ ≥100 Gy (Fig. 3) were significant prognostic factors for DMFS, LRFS, PFS and OS. If tolerated by normal tissue, BED_10_ ≥100 Gy was also recognized as radiation “ablation” at other sites [27]. The 5-year OS was 55.8 % in BED10 ≥100 groups based on the findings in our study. However, the lesions with cumulative GTV >5 cm could be caused by the application of a non-ablative dose (BED_10_ <100 Gy). Based on the findings in our study, EQD_2_ ≥74 Gy may be recommended as the “underscore” for a second-line scheme, ﻿the 5-year OS was 48 %. And EQD_2_ <74 Gy may be recommended as palliative irradiation, the 4-year OS was 28.1 %. If tolerated by normal tissues, BED_10_ ≥100 Gy and EQD_2_ ≥74 Gy should receive more attention when the SBRT plan is designed.

**Fig. 4:**
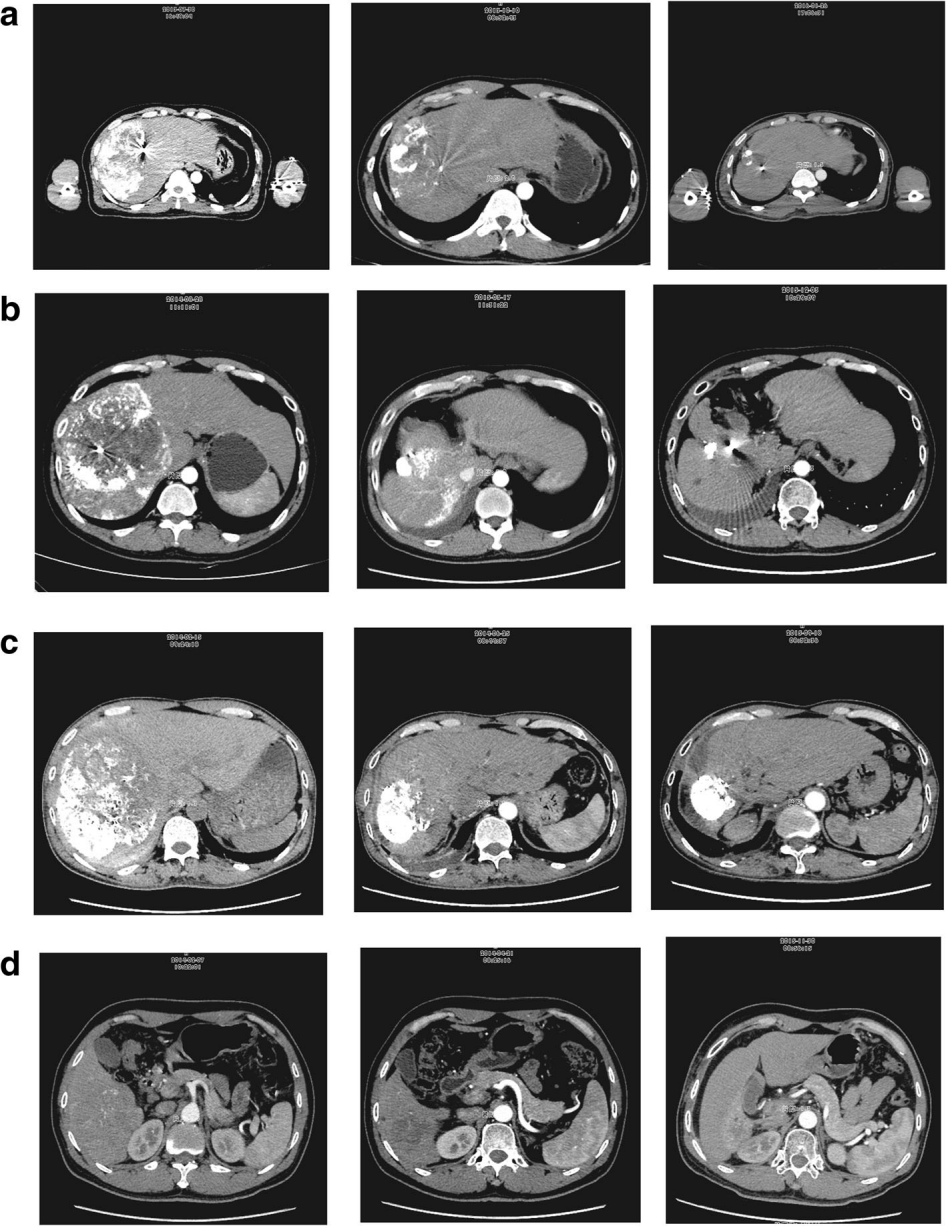
SBRT can result in a complete response in huge HCCs. **a** TAE + SBRT; **b** TAE + SBRT; **c** TAE + SBRT; **d** SBRT only

Que et al. reported the findings of 22 patients with huge unresectable HCCs (≥10 cm) treated with SBRT at a dose range of 26–40 Gy in 5 fractions. The 1-year OS was 50 % with a median survival of 11 months [28]. In the current study, univariate analysis showed that tumor size was a significant prognostic factor for OS. Thirty-three patients with huge HCCs were treated with SBRT. The 1-, and 3-year OS was 51.8 and 38.3 %, respectively, and SBRT achieved substantial tumor regression (Fig. 4).

A total of 68 patients received TAE in our hospital and 9 patients received TACE at another hospital. The toxicity and side effects in the TAE/TACE + SBRT group and SBRT group were limited (Table 4). RILD was observed in one CTP A patient and two CTP B patients.

This study has several limitations. First, this was a retrospective, non-randomized single-center study. Second, in the in-depth comparative analysis of TAE/TACE + SBRT and SBRT alone, only a few patients had CTP class B in the TAE/TACE + SBRT group compared with the SBRT group (9 % versus 18.0 %, *P* = 0.139), BED_10_ ≥100 Gy was lower in the TAE/TACE + SBRT group than in the SBRT group (31.2 % versus 42.0 %, *P* = 0.031) and the percentage of patients with tumor size ≥10 cm was higher in the TAE/TACE + SBRT group than in the SBRT group (31.0 % versus 16.3 %, *P* = 0.013). The statistical strength may have decreased and selection bias may have increased. Third, the follow-up period in the entire patient population was short, which could have obscured late effects. However, TAE/TACE + SBRT may be recommended as a treatment for unresectable HCC >5 cm. Further multi-institutional prospective studies are warranted to investigate the true effect of this novel treatment.

## Conclusion

SBRT combined with TAE/TACE may be an effective complementary treatment approach for HCC >5 cm. BED_10_ ≥100 Gy and EQD_2_ ≥74 Gy should receive more attention when the SBRT plan is designed.

## Abbreviations

AFP: Alpha fetoprotein
ALT: Alanine aminotransferase
AST: Aspartate aminotransferase
BCLC: Barcelona clinic liver cancer
BED: Biologically effective dose
CT: Computed tomography
CTP: Child-Turcotte-Pugh
DMFS: Distant metastasis-free survival
ECOG: Eastern cooperative oncology group
EQD_2_: Equivalent dose in 2 Gy fraction
GTV: Gross tumor volume
HCC: Hepatocellular carcinoma
LC: Local control rate
LRFS: Local relapse-free survival
MRI: Magnetic resonance imaging
NCI CTCAE: Common terminology criteria for adverse events
OS: Overall survival
*P*: *P*-value
PFS: Progression-free survival
PTV: Planning target volume
RECIST: Response evaluation criteria in solid tumors
RILD: Radiation-induced liver disease
RT: Radiotherapy
SBRT: Stereotactic body radiation therapy
TAE/TACE: Trans-arterial embolization
